# Treatment of aggression regulation problems with virtual reality: study protocol for a randomized controlled trial

**DOI:** 10.3389/fpsyg.2024.1324644

**Published:** 2024-04-04

**Authors:** Bas R. van Wolffelaar, Joan E. van Horn, Larissa M. Hoogsteder

**Affiliations:** ^1^De Forensische Zorgspecialisten, de Waag, Utrecht, Netherlands; ^2^Program Group: Forensic Child and Youth Care, University of Amsterdam, Amsterdam, Netherlands

**Keywords:** virtual reality, VR, responsive aggression regulation therapy, Re-ART, aggression, forensic outpatients, forensic psychiatry, randomized controlled trial

## Abstract

**Background:**

Aggressive conduct among delinquents presents a pervasive issue, bearing substantial implications for not only society at large but also for the victims and the individuals displaying the aggression. Traditional approaches to treating aggression regulation deficiencies generally employ Cognitive Behavioral Therapy (CBT) in conjunction with analog role-playing exercises. A body of research supports the efficacy of various therapeutic models for aggression regulation, including Responsive Aggression Regulation Therapy (Re-ART). Role-playing within a therapeutic context has been shown to contribute significantly to reductions in violent reoffending. However, the practical application of these skills in real-world settings remains challenging due to the inherent risk of aggressive outbreaks. Additionally, the conventional role-playing scenarios, often conducted in a therapy room, lack contextual realism and may induce role confusion between the patient and the therapist. Virtual Reality (VR) technology could offer a viable solution to these limitations by allowing for skill training in both behavioral and cognitive domains within a realistic yet safe and controlled setting. The technology also facilitates real-time awareness of emotional states and tension levels in the patient. This paper describes the study protocol of a randomized controlled trial in which Re-ART offered in a virtual environment (Re-ART VR) is compared to Re-ART offered as treatment as usual.

**Methods and analysis:**

Adult forensic outpatients with aggression regulation problems are randomly assigned to either Re-ART VR or Re-ART. The *Controlling Skills*, *Influence of Thinking* and *Handling Conflicts* modules will be offered to both groups during 3–6 months. Pre- and post-intervention measurements are performed. The primary outcome measurement is the degree of aggression regulation, while secondary outcome measurements include impulsivity and cognitive biases. Additionally, patient motivation and therapist motivation are expected to act as moderating factors.

**Discussion:**

To date, scarcely previous research has been done on the effectiveness of VR in treatment of aggression regulation problems in forensic outpatients. Forensic outpatients who do not benefit sufficiently from mainly CBT-based interventions may benefit more from experiential learning. The unique capabilities of VR in this regard have the potential to enhance the treatment effect.

**Clinical trial registration**: [https://clinicaltrials.gov/], identifier [NL78265.018.21].

## 1 Introduction

Aggressive behavior poses a significant challenge to society, impacting not only the individuals involved but also the wider community. Despite a decline in international crime rates among young adult offenders (Berghuis and De Waard, 2017; Fernández-Molina and Bartolomé Gutiérrez, 2020), the persistence of violent behaviors like threats, abuse, vandalism, and public order disturbances remains a major concern due to their profound consequences on both victims and society (e.g., Aarten et al., 2020). Violent behavior has significant financial implications, affecting various aspects such as victims’ costs and criminal justice expenditures (Cohen and Piquero, 2015), and legal financial obligations for offenders (Pleggenkuhle, 2018). Moreover, violent offenders often exhibit high rates of recidivism, perpetuating the cycle of harm (Falk et al., 2014; Alper et al., 2018; Weijters et al., 2018; Stewart et al., 2019). Improving therapy for violent offenders is essential. Research indicates that integrating elements of Cognitive Behavioral Therapy (CBT) with Virtual Reality (VR) holds promise. For this reason, this study focuses on the impact of VR in treatment of aggression regulation problems.

The primary approach to treating aggression regulation problems involves CBT. Overall, research highlights the significant impact of interventions incorporating role-playing, cognitive skills, and homework assignments in treatment, leading to notable decreases in general and/or violent re-offending compared to interventions lacking these elements (Jolliffe and Farrington, 2007; Papalia et al., 2019; Hoogsteder et al., 2023a). Responsive Aggression Regulation Therapy (Re-ART) is an example of an aggression regulation intervention that combines CBT-elements with role-playing and experiential exercises such as chair techniques, imaginations, and mindfulness exercises (Hoogsteder and Bogaerts, 2018). Initially, Re-ART was developed for boys and girls aged 16–21 years in residential care with severe aggression regulation problems and a moderate to (very) high recidivism risk. Its effectiveness is now demonstrated in young adults in both in- and outpatient forensic treatment (Hoogsteder et al., 2014a,b; Schippers et al., 2020). More specifically, compared to control groups receiving treatment as usual, Re-ART showed to improve coping skills, treatment motivation, and diminishes impulsivity and cognitive distortions, leading to a significant reduction in both violent and general recidivism after 2 and 3 years (Hoogsteder et al., 2018, 2021). By integrating evidence-based elements, Re-ART aims to provide a promising approach to address and manage aggressive delinquent behavior. However, despite these positive outcomes, the effect of aggression regulation therapy remains moderate, indicating the need for further refinements in treatment strategies (Papalia et al., 2020; McIntosh et al., 2021).

A new technology that seems promising in increasing the treatment effects of role playing, among other things, is VR. Role-playing in VR presents distinct advantages, allowing controlled exposure to stimuli that might be too risky or harmful in real-life situations, thereby safeguarding others. Moreover, the use of avatars in VR role play eliminates role confusion that can occur especially when therapists engage in so-called analog role-play with their patients (Fromberger et al., 2014). VR is valued for its ability to facilitate implicit and experiential learning (Slater and Sanchez-Vives, 2016; Kip et al., 2018). Comparative studies, such as that by Park et al. (2011), indicate that VR role-playing outperforms analog role-playing in enhancing conversational skills, assertiveness, treatment interest and skill generalization. Additionally, VR requires active engagement from therapists and patients. This is expected to boost patient motivation and healthcare professional enthusiasm, potentially enhancing therapeutic relationships and treatment effectiveness (Kip et al., 2019a,b).

Previous research investigating the application of VR in treating mental disorders has demonstrated its effectiveness for specific phobias, as well as other clinical conditions such as obsessive-compulsive disorder, eating disorders, autism, schizophrenia, and post-traumatic stress disorder (Eichenberg and Wolters, 2012; Turner and Casey, 2014; Valmaggia et al., 2016; Freeman et al., 2017; Wiebe et al., 2022). While it is generally acknowledged that VR shows promise as a valuable addition to existing treatments, further research is required to fully understand its effectiveness. In the context of forensic treatment, the use of VR in forensic mental health care is expected to have added value (Kip et al., 2019a,b; Roggeman et al., 2021), but research is still in its early stages. Evidence suggests the effectiveness of applying VR in forensic setting (Jo et al., 2022; Johnston et al., 2023). Findings from an initial randomized controlled trial indicated that an intervention that integrated VR with serious gaming did not yield superior results in reducing anger and aggressive behavior compared to the control condition among forensic psychiatric outpatients with aggression regulation issues. Nevertheless, qualitative data revealed that participants reported gaining more insight into their own behavior and the behavior of others (Smeijers et al., 2021). As far as our knowledge extends, no other studies have yet been conducted on the effectiveness of VR in treating aggression regulation problems within an outpatient forensic setting. Thus, more research is needed to explore its potential benefits in this specific domain (Sygel and Wallinius, 2021).

In forensic inpatient studies involving VR, positive pre-post effects were observed on self-reported direct aggression and hostility, anger control and impulsiveness compared to a waiting list control group. However, these effects ceased to exist after a 3-month follow-up period (Klein Tuente et al., 2020). The authors put forth several explanations for these findings, with the closed setting being the most significant factor. The confined environment made it challenging to apply the skills learned in the VR setting to real-life situations effectively. Moreover, the patients were not actively encouraged to practice their newly acquired skills between sessions or given homework assignments. Furthermore, the research protocol lacked options for personalized interventions, as advocated in the Risk-Need-responsivity model (RNR), the most applied rehabilitation model in forensic treatments (Andrews and Bonta, 2017). These limitations likely contributed to the diminished medium-term impact of the VR intervention. To the best of our knowledge, no studies have been conducted to compare inpatient and outpatient groups regarding the transfer of acquired skills into practical application. However, one could hypothesize that the effectiveness of VR within an outpatient setting may be higher due to the increased opportunity for patients to practice and apply the learned skills in their daily lives. This is especially true considering that homework assignments are a standard component of the Re-ART treatment.

The primary objective of this Randomized Controlled Trial (RCT) is to assess the potential benefits of incorporating VR into the existing Re-ART. More specifically, the study aims to examine the extent to which VR enhances the degree of aggression regulation among aggressive forensic outpatients (Re-ART VR) above and beyond the effects found in their counterparts receiving the regular Re-ART intervention. The effect of cognitive biases and impulsivity as determinants of aggressive behavior is examined as secondary outcome measures as well. Moreover, the study will explore motivation as a moderating variable, both among patients undergoing treatment and among the therapists delivering the intervention. The hypothesis posits that patients treated with Re-ART VR will experience greater enhancements in the degree of aggression regulation, a more substantial reduction in cognitive biases and impulsivity compared to those receiving regular Re-ART. Additionally, it is expected that higher levels of patient motivation and therapist motivation will contribute to more pronounced improvements.

## 2 Methods and analysis

### 2.1 Study design

**Figure 1:**
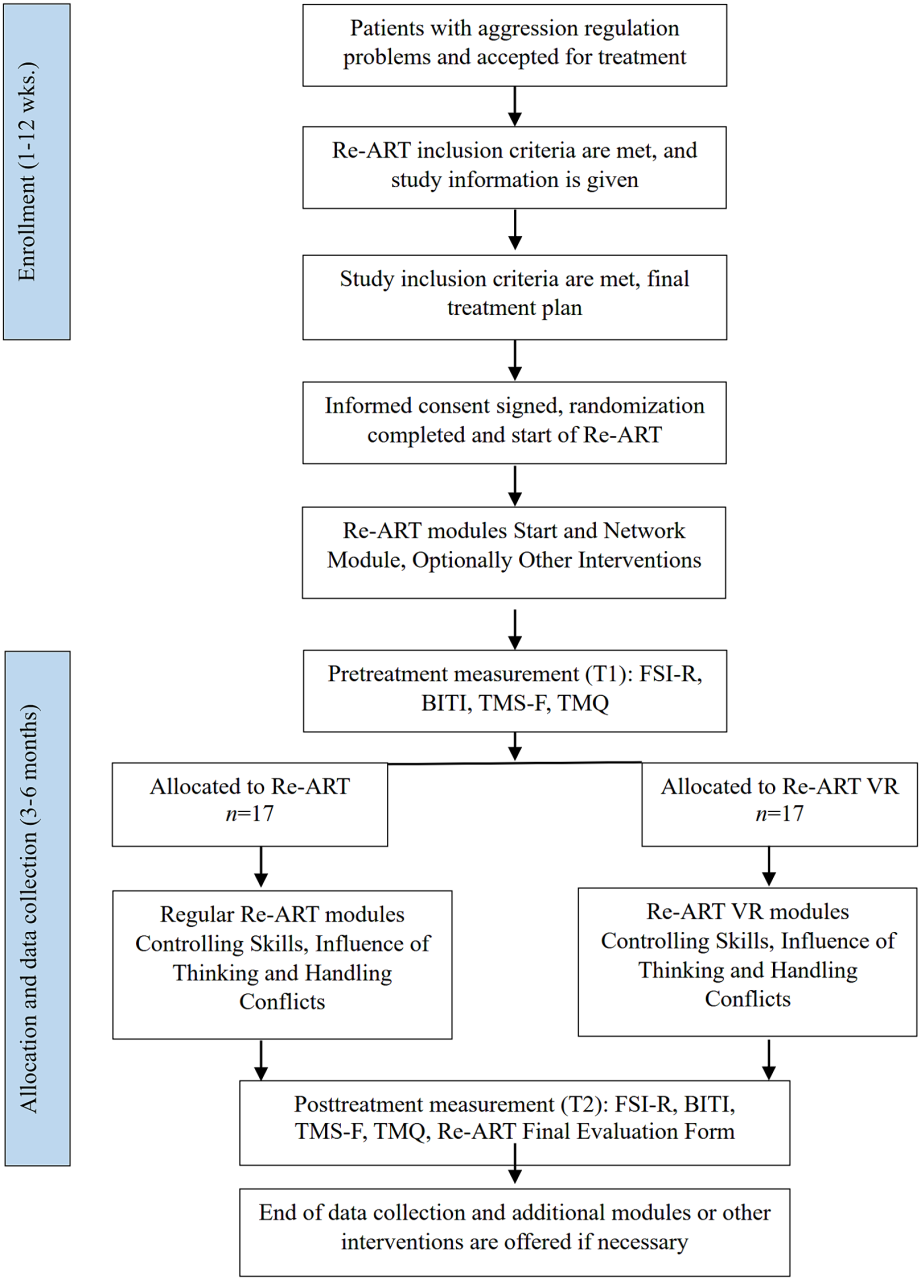
Flow diagram.

An RCT with a pretest-posttest design will be carried out in which forensic outpatient with aggression regulation problems are being assigned to either Re-ART using VR (Re-ART VR) or regular Re-ART. Pre- (prior to the Controlling Skills module—T1) and post-intervention (at the end of the Handling Conflicts module—T2) measurements are performed. Figure 1 presents the flow diagram.

### 2.2 Setting

The study will be conducted at a large Dutch center for forensic outpatient treatment. Mainly CBT-based interventions are offered to juvenile and adult offenders who, due to their offense behavior, (are prone to) encounter police force or judicial authorities. Offense behaviors can vary from aggression in or outside the family, property offenses with or without violence, or sexual offenses. Several general exclusion criteria for treatment - clinically assessed at the registration and intake phase - are applicable. General exclusion criteria for treatment are acute psychosis and serious addiction problems that require supervised detoxification, due to the absence of the specific prerequisites at the treatment center for addressing these issues. Patients enter treatment on a voluntary or mandatory basis. In both scenarios, the patients participating in the study meet the eligibility criteria for Re-ART. Voluntary treatment indicates that the patient enters treatment on his own initiative, on referral of a general practitioner or another mental health care institute. Mandatory treatment means that treatment is imposed by a judge. In these cases, a probation officer fulfills the supervisory role.

### 2.3 Participants

The study includes adult (18 years or older) male patients who meet the inclusion criteria for Re-ART, which entails exhibiting aggressive behavior and having a moderate to high risk of recidivism. The study takes place at four out of the thirteen locations of the treatment center, as these four locations are equipped with VR sets. Therapists receive training in CleVR’s VR-CBT software (see section 2.6.4.1), either from a CleVR trainer or from colleagues with VR experience, gaining extensive experience through their regular clinical practice. The main researcher provides instructional materials, including videos demonstrating VR exercises.

#### 2.3.1 Sample size

To ensure sufficient statistical power for detecting significant effects in our repeated measures ANCOVA with within-between interaction, an *a priori* power analysis was conducted (G*Power; Faul et al., 2009). Using an expected effect size (f) of 0.25, a significance level (α) of 0.05, and a desired power (1-β) of 0.80, the calculation revealed that a total sample size of 34 participants is required for the study (17 in each condition). The analysis assumes two groups (Re-ART VR vs. regular Re-ART) with two measurement points (pre- and post-intervention), a correlation of 0.5 among repeated measures, and no sphericity correction (ε = 1).

#### 2.3.2 Recruitment

Therapists will approach patients who meet the study inclusion criteria (see Figure 1). Patients who meet the Re-ART inclusion criteria (e.g., moderate to high recidivism risk, poor impulse control) are considered for study inclusion, except for those who receive concurrent interventions (e.g., trauma-focused therapy or treatment of substance abuse) during the study. This is done to prevent potential interference with the efficacy measurements in the current study. Following a comprehensive oral and written explanation of the study, patients who meet the study inclusion criteria (adult, male) will be requested to provide informed consent during discussion of the final treatment plan. Patients will be offered a visit to the VR-room to familiarize themselves with what they can anticipate in the VR-condition. They can also contact the first author or the independent expert for more information. After the treatment plan is discussed with the patient, interventions that are indicated can take place prior to the start of the study (e.g., Re-ART module Stress Reduction, trauma-focused therapy). Pretreatment measurement takes place just before start of the study (Controlling Skills module). Upon finishing the questionnaires and forwarding them to the main researcher, patients will be informed about the condition they are assigned to.

#### 2.3.3 Randomization

After obtaining written consent from the patient, the first author randomizes included patients using the online randomization program available at https://www.graphpad.com/quickcalcs/randomize2. Each time the program is utilized, it generates a distinct randomization list based on the date and time of usage. The randomization list will be accessible exclusively to the researchers.

### 2.4 Interventions

#### 2.4.1 Responsive aggression regulation therapy

Re-ART is a protocolized intervention designed for (young) adults facing significant challenges with emotional and/or instrumental aggression regulation, and who exhibit a moderate to high recidivism risk. Incorporating the RNR principles (Andrews and Bonta, 2017), Re-ART demonstrates its flexibility by adapting the duration of the intervention (individual and group modules) according to the risk level, ranging from half a year to approximately 2 years (Risk principle). Additionally, it customizes treatment components based on the individual’s specific criminogenic issues (Needs principle), and tailors the intervention to the patient’s intelligence, learning style, pace, and preferred learning methods, among other factors (Responsivity principle).

The Re-ART treatment comprises standard modules, including Start, Network, Controlling Skills and Influence of Thinking. Optional modules, such as Stress Reduction, Impulse Control, Emotion Regulation, Observation and Interpretation, Self-Image and module for family systems, offer additional customization.

A comprehensive theoretical and program manual (Hoogsteder and Bogaerts, 2018) provide guidelines for each module, detailing the exercises and components to be included, along with the specific objectives that must be accomplished before progressing to the subsequent module. Moreover, the manuals outline the effective therapeutic techniques aimed at diminishing undesirable behaviors and fostering more desirable behaviors. These include motivational techniques to bolster patients’ self-belief and motivation to change, the aggression chain, psychoeducation to raise awareness of the negative consequences of aggressive behavior, exercises to manage and reduce negative emotions and interventions to identify and change irrational thoughts. The intervention also encourages patients to consider alternate perspectives by adopting the viewpoint of others with more facilitative ways of thinking.

Patient engagement in Re-ART consists of individual sessions occurring at least once a week, lasting for a minimum of 1 h. Depending on the risk level, more sessions per week can take place. Group training can take place, but the focus predominantly lies on individual treatment.

The current study focuses on measuring the effect of the Controlling Skills, Influence of Thinking and Handling Conflicts modules, which are offered in that order and usually last about 3–6 months. The Controlling Skills module focuses on learning control methods, to help individuals take a time-out more easily or stay calm when a time-out is not feasible, and to apply these methods in practice. The Influence of Thinking module focuses on reducing distorting cognitions and applying helping thoughts during difficult situations. The Handling Conflicts module aims to teach various skills necessary to handle conflict constructively, such as communicating appropriately, dealing with authorities and dealing with criticism.

#### 2.4.2 Responsive aggression regulation therapy with virtual reality

In the Re-ART VR condition, identical modules (Controlling Skills, Influence of Thinking and Handling Conflicts) with the same exercises and role plays are provided, utilizing VR. For example, situations that provoke anger are simulated, allowing patients to practice skills aimed at preventing the escalation of aggression (e.g., applying a helpful thought or responding assertively rather than aggressively). In addition to exercises and role plays, the modules encompass various components, including psycho-education, mapping triggers, identifying dysfunctional cognitions and exploring conflict styles. To ensure that in the experimental condition VR is a substantial part of the treatment, it was determined that a minimum of 60 or 66% (depending on the module) of the sessions per module should be offered with VR. To monitor the attainment of this percentage, therapists fill out the Program Integrity checklist (see section 2.6.4.2).

Following the completion of the study, additional modules, including Impulse Control, Observation and Interpretation, Emotion Regulation and the Self-image module, can be made available if deemed necessary. This provision becomes applicable after the post-test measurement is conducted and extends to other interventions as well, such as pharmacotherapy and treatment of substance abuse. Moreover, the therapist, in collaboration with the patient, then has the flexibility to opt for VR treatment, irrespective of the study condition in which the patient was during the study.

### 2.5 Criteria for discontinuing study participation

Participants who decide to discontinue participation in the study will receive a follow-up from their therapist to understand the reasons for their withdrawal. They will also have the option to be contacted directly by the main researcher. Feedback regarding the withdrawal will be sent via email to the main researcher to categorize responses for future discussion or reference. If any participant reports experiencing adverse effects or feels negatively impacted by their participation, they will be invited to a face-to-face meeting with the main researcher, therapist, and if necessary, a team leader. Additionally, participation in the study will be discontinued if, due to unforeseen circumstances during the study period, another intervention besides the three Re-ART modules must be provided.

#### 2.5.1 Cyber sickness

Cyber sickness, also known as VR sickness or simulator sickness, is a common issue experienced by some individuals while using VR technology (Weech et al., 2019). Symptoms are like those of motion sickness, but they are less severe and less common (Kennedy et al., 1993). Cyber sickness occurs when there is a disconnect between the visual and vestibular systems, leading to sensory conflict and discomfort. Symptoms of cyber sickness include nausea or vomiting, dizziness, tired eyes, disorientation, dry mouth, sweating and problems with balance (LaViola, 2000; Davis et al., 2014). Some studies suggest that symptoms (at least partially) can be explained by overlap with anxiety symptoms (Fornells-Ambrojo et al., 2008). Therefore, it is expected that cyber sickness symptoms will decrease as anxiety symptoms decrease (Pot-Kolder et al., 2018). In case patients experience symptoms, such as dizziness or nausea, due to exposure to the VR environment, the therapist will help alleviate their discomfort. Several actions can be taken to prevent cyber sickness, such as building up the time a participant spends in the VR environment gradually and instruct the participant not to move his/her head too fast and in particular to walk straight (Rebenitsch, 2015). The main researcher provides a cyber sickness protocol, including instructions and strategies for therapists to prevent symptoms of cyber sickness. If cyber sickness symptoms persist and do not improve, the VR session will be halted, and a joint evaluation with the patient will determine the feasibility of continuing VR therapy. Should continuing VR therapy not be possible, the patient will be excluded from the study. Participants will be advised to seek a medical check-up with their general practitioner if any medical condition arises during the intervention period.

### 2.6 Materials

For an overview of the measurements (see Figure 1).

#### 2.6.1 Primary outcome measurement

##### 2.6.1.1 Degree of aggression regulation

The Forensic Symptoms Inventory-Revised Adults (FSI-R) (Van Horn, 2017) will be used to measure changes in the degree of aggression regulation. This self-report questionnaire consists of 32 items measuring eight domains of which a combined total score serves as a measure for the degree of aggression regulation: Aggression (e.g., “I threatened others”) and Anger (e.g., “I was annoyed”). Each subscale consists of four items, resulting in a combined scale of eight items that are rated on a 5-point scale ranging from 1 “(almost) never” to 5 “(almost) always.”

Higher scores on the subscales are indicative of increased deficits in cognitive, behavioral, and/or emotional functioning. The Aggression subscale assesses the extent of physical and verbal aggression. Patients with a high score on this subscale struggle to effectively manage their feelings of anger and rage, resulting in physical and/or verbal violence toward objects and/or individuals. The Anger subscale evaluates elevated levels of both direct and underlying anger. Patients with high anger scores may face an increased risk of aggressive impulse breakthroughs.

Multi-Group Confirmatory Factor Analyses supported the measurement and structural invariance with respect to gender and age groups (18–23 years and ≥ 24 years) (Van Horn, 2018) as well as longitudinal measurement invariance (Ten Hag, 2016). Reliability coefficients were in the acceptable to good range (α ≥ 0.68) for all subscales (Ten Hag, 2016; Mac Intosh, 2021).

#### 2.6.2 Secondary outcome measurement

##### 2.6.2.1 Cognitive biases

To assess cognitive biases, the Brief Irrational Thoughts Inventory (BITI) will be used (Hoogsteder et al., 2014b). The BITI is a self-report questionnaire comprising 16 statements that capture three types of irrational thoughts: Aggression and Justification (9 items, e.g., “If someone touches me, I should hit him”), Sub-Assertiveness (4 items, e.g., “I think that people get angry” with me because I often say “No”), and Distrust (3 items, e.g., “Everyone is against me”). Respondents rate each item on a six-point Likert scale, ranging from 1 “totally disagree” to 6 “totally agree.” The BITI’s construct validity is supported by convergent, divergent and concurrent validity evidence. Furthermore, measurement invariance was established across gender and ethnic origin (native versus non-native Dutch respondents) through confirmatory factor analysis, confirming the robustness of the BITI as a valid measure (Hoogsteder et al., 2014b). The list appears to be useful for adults as well. The BITI was also found to be measurement invariant for the background characteristics of age, sex, intellectual disability and migration background (Schoute, 2022).

##### 2.6.2.2 Impulsivity

From the FSI-R, the impulsivity subscale will be used to measure a general predisposition toward rapid, unplanned emotional (e.g., diminished ability to delay gratification) or behavioral (e.g., acting on the spur of the moment) reactions to internal or external stimuli without thinking about the consequences. The subscale contains four items (e.g., “I longed for more excitement”), each scored on a 5-point scale ranging from 1 “(almost) never” to 5 “(almost) always.” For reliability and validity data of the FSI-R, see section 2.6.1.1.

FSI-R and BITI are part of the standard Routine Outcome procedure in the forensic outpatient treatment center. Routine assessments of the FSI-R and BITI, which are typically conducted every 3–4 months, will sometimes be omitted if they closely coincided with pre- or post-treatment measurements. This is done to lessen the burden on patients participating in the study.

#### 2.6.3 Moderators

##### 2.6.3.1 Patient motivation

Patient motivation is assessed using the Motivation to Engage in Treatment (MET) scale of the Treatment Motivational Scales for forensic outpatient treatment (TMS-F) developed by Drieschner and Boomsma (2008a). This self-report questionnaire comprises 85 statements designed to gauge the motivation of forensic patients for engaging in outpatient treatment. Beside the MET scale, the TMS-F encompasses six Internal Determinants (ID) scales and a social desirability scale. The MET scale serves as a measure for Patient Motivation, Drieschner and Boomsma (2008b) conclusion that in particular the MET scale predicts treatment engagement to a substantial degree. The MET scale contains 16 items (e.g., “I would stop therapy if I see no change in my life”), each scored on a 5-point scale ranging from 1 “totally disagree” to 5 “totally agree.” Drieschner and Boomsma (2008a) found that the MET scale demonstrate adequate internal consistency, with the sum score of the items being reliably measured (*α* = 0.88) for most purposes.

##### 2.6.3.2 Therapist motivation

Therapist motivation is measured with a self-constructed questionnaire, the Therapist Motivation Questionnaire (TMQ). The TMQ consists of 13 items (e.g., “I experience positive emotions while giving treatment sessions to my patient”) pertaining to various aspects, such as experiencing a positive relationship with the patient, having self-assurance in one’s abilities, exerting effort to assist the patient with their issues and actively involving the patient in the treatment process. Answering categories vary on a 6-point scale from 1 “totally disagree” to 6 “totally agree.” High scores reflect increased motivation.

#### 2.6.4 Other materials and measurements

##### 2.6.4.1 Virtual reality software

The VR-CBT software utilized in this study was collaboratively developed by CleVR^1^ in conjunction with researchers from various academic institutions, mental health facilities, child and adolescent psychiatry and forensic psychiatry. CleVR’s VR-CBT software has a CE mark of quality as a medical device and can be safely used as a medical aid within (mental) healthcare. The VR-CBT software enables the creation of personalized scenarios and interactive virtual (social) worlds.

Accompanied by a comprehensive manual (Social Worlds 4.1), the VR software empowers the therapist to govern the virtual environment through a laptop. During the VR session, the patient wears Oculus Rift VR glasses (type S, v1.2), fully encompassing their field of view and allowing complete immersion in the virtual environment. Concurrently, the therapist observes the same virtual setting on a computer screen, facilitating seamless communication with the patient via headphones connected to a microphone. This control encompasses adjusting characters’ behavior within the virtual realm, including actions like approaching, arm movements and gaze, as well as regulating their emotional expressions, such as displaying anger. To further enhance the immersive experience, a dual-joystick configuration allows the patient to navigate within the virtual environment while simultaneously providing real-time visual feedback of hand movements in the simulated world. Moreover, the therapist can manipulate the characters’ voices by utilizing a voice shaper integrated with the MorphVox Pro program.^2^

**Figure 2:**
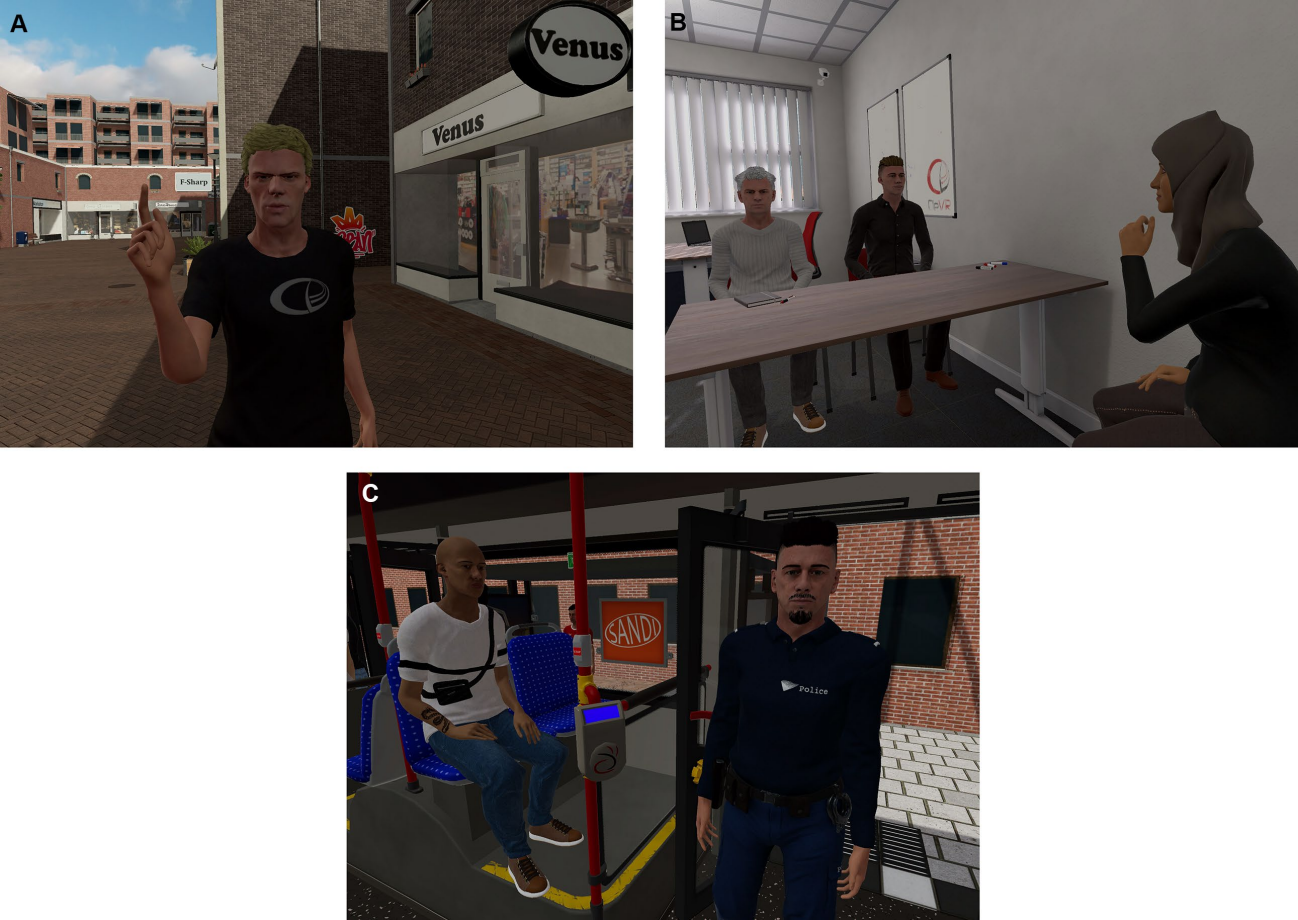
Virtual environments: shopping street **(A)**, office **(B)**, and bus **(C)**. Source: CleVR B.V., Delft, the Netherlands.

The VR session begins as the patient put on the VR glasses and headphones, and if needed, holds the joysticks. At the outset, the patient is introduced to a “virtual waiting room,” which serves as a neutral and low-stimulation environment, allowing them to adapt to the VR setting. Once the patient feels at ease, the therapist proceeds to load the targeted VR environment, and the role-playing or exercise commences. Importantly, throughout the VR session, the therapist retains the ability to “pause” the experience, accomplishing this by deactivating the voice modifier and directly communicating with the patient using their own voice within the virtual environment. Figure 2 shows some examples of virtual environments.

##### 2.6.4.2 Program integrity

To check whether the study protocol in both conditions has been carried out according to the design, the therapist keeps a Program Integrity checklist in which the therapist records several aspects for each module, including the number of (analog or VR) role-play exercises conducted per session, the total sessions with and without role-play and whether the patient engaged in the role-plays in both a relatively relaxed state and a tense state, among other factors. In addition, the therapist is asked to report all details, including whether and why deviations from the protocol occurred and whether other therapy sessions were conducted that were not prescribed in the module.

##### 2.6.4.3 Feedback on study protocol

To gather feedback on the study protocol, the Re-ART final evaluation form is employed. This form is a standard part of the Re-ART treatment, with versions for both patients and therapists. For the purposes of this study, modifications have been made to the forms. Questions related to feasibility and usability were added, while questions not relevant to the study were removed. Instead of administering the forms at the end of the Re-ART treatment, they will be given at the conclusion of the study.

### 2.7 Data management

Data relevant to the study will be stored in a distinct research file, accessible only to the researchers involved in the study. To ensure security, SPSS files will be stored in a protected Citrix environment. During data entry into SPSS, researchers will use unique codes to differentiate subjects. These codes can be cross-referenced with a separate password protected Excel spreadsheet to identify individual subjects. The linkage between subject codes and patient files will be exclusively accessible to the researcher team, maintaining confidentiality and data integrity.

#### 2.7.1 Statistical analyses

The data analysis will be conducted using IBM SPSS v29. The analytical plan aims to examine the effectiveness of Re-ART with VR (Re-ART VR) compared to standard Re-ART in a forensic outpatient setting. Intention to Treat (ITT) analyses as well as Per Protocol (PP) analyses will be conducted. The ITT analyses will include data from all randomized patients, regardless of drop-out, whereas PP analyses will include only those patients who completed the treatment as originally allocated.

To evaluate treatment effects on primary and secondary outcomes, a Repeated Measures Analysis of Covariance (RM-ANCOVA) will be performed. This approach allows to account for both baseline scores and time, along with other potential covariates, thus providing a more precise estimation of the intervention effect.

##### 2.7.1.1 Preliminary analyses

Before conducting the main analyses, the data will be inspected for missing values (e.g., MCAR—Missing Completely At Random), outliers, and the assumptions of RM-ANCOVA, including sphericity, homogeneity of variances, and linearity. Missing data will be addressed using multiple imputations (Expectation Maximization—EM), and outliers will be examined and treated to ensure they do not bias the results.

To verify the randomization results, various background variables such as age, migration background, and primary diagnosis will be compared between patients in Re-ART VR and regular Re-ART using chi-square tests for dichotomous measures and independent *t*-tests for continuous measures. These variables will be included as covariates in the RM-ANCOVA if significant differences are found.

##### 2.7.1.2 Primary outcome analysis: degree of aggression regulation

An RM-ANCOVA will be conducted with the FSI-R scores at both pre- and post-intervention as the within-subjects factor. The treatment condition (Re-ART VR vs. regular Re-ART) will serve as the between-subjects factor. Covariates like age, motivation level, and risk of recidivism will be included to control for initial differences.

The primary focus will be on the interaction term between treatment condition and time to examine whether the rate of improvement in aggression regulation differs between the two conditions.

##### 2.7.1.3 Secondary outcome analysis: cognitive biases and impulsivity

For secondary outcomes, separate RM-ANCOVA’s will be conducted for the BITI and the Impulsivity subscale of the FSI-R, using pre- and post-intervention scores as the within-subjects factors and treatment condition as the between-subjects factor. Additional covariates will be included as controls.

##### 2.7.1.4 Moderators: patient and therapist motivation

To examine whether patient and therapist motivation moderate treatment outcomes, interaction terms between treatment condition and the respective motivation scales (MET scale of the TMS-F for patients and TMQ for therapists) will be included in the RM-ANCOVA models.

##### 2.7.1.5 Sensitivity analyses

To assess the robustness of the results, sensitivity analyses will be performed to determine how the outcomes change when different covariates are included or excluded.

##### 2.7.1.6 Correction for multiple comparisons

Given the multiple analyses conducted in this study, a correction for multiple comparisons will be applied to control the Type I error rate. We will use the Bonferroni correction method, which will adjust the alpha level accordingly.

## 3 Discussion

The main goal of this randomized controlled study is to evaluate the potential advantages of integrating VR into the existing Re-ART. VR is a relatively new technology in forensic outpatient treatment and preliminary evidence on its effectiveness has been provided only in forensic clinical samples (Klein Tuente et al., 2020). Based on the possibilities offered by VR (including being able to safely practice social skills in a role-playing game) and the opportunities to practice the learned skill in their own environment, it is plausible to assume that offering VR to forensic outpatients in addition to regular therapy will increase the treatment effect. To this end, an RCT-study has been set up to provide evidence for the added value of VR in enhancing aggression regulation skills in aggressive forensic outpatients (Re-ART VR) beyond the effects observed in those receiving the standard Re-ART intervention. Additionally, the research will explore motivation as a moderating factor, both among patients undergoing treatment and the therapists providing the intervention. It is expected that patients in the Re-ART VR-group will demonstrate a more significant improvement during the second assessment compared to patients in the regular condition, especially if both patients and therapists exhibit higher motivation levels. Including motivation as a moderator is a valuable addition because research shows that the lack of motivation in patients increases the chance of dropout, which in forensic patients eventually increases the chance of re-offending (Norcross et al., 2011).

### 3.1 Study strengths

The randomized controlled trial (RCT) as described in the study protocol offers several strengths, chief among them the robustness inherent to its design. The RCT method is a gold standard in intervention research, providing a high level of internal validity. Another strength is the incorporation of VR as a novel modality for delivering Re-ART. Given the immersive capabilities of VR, this study could pave the way for more engaging and effective treatment regimes in forensic outpatients, a population that typically presents complex treatment needs. The use of VR also allows for a controlled, safe environment where patients can practice skills that would otherwise be risky to rehearse in real-life settings. Additionally, the study employs multiple outcome measures, including assessments of the degree of aggression regulation, cognitive biases and impulsivity, thereby providing a comprehensive evaluation of treatment effectiveness. The inclusion of both patient and therapist motivation as moderating variables is another crucial strength, recognizing the role that these factors play in treatment success.

### 3.2 Potential limitations

While the study is designed with rigor, it is not without limitations. One limitation could be the sample size. Although power analyses suggest that 34 subjects will be adequate, this number may not be large enough to detect small but clinically significant differences between the two treatment modalities. This is particularly the case because the study focuses on a component of treatment rather than the entire intervention, and the issues faced by the target group are intractable. Another limitation is the potential for a novelty effect with the VR condition. Patients may initially be more motivated or engaged simply because the VR treatment is new and different, and this could potentially confound the results (Klein Tuente et al., 2020). Additionally, the study’s focus on adult male participants may limit the generalizability of the findings to broader forensic populations, such as females or adolescents. Furthermore, the limited duration of the intervention (3–6 months) and absence of follow-up measurement raises questions about the long-term sustainability of any observed treatment gains.

### 3.3 Challenges

A key challenge is ensuring treatment fidelity across both the standard Re-ART and Re-ART VR conditions. Maintaining the integrity of the interventions and controlling for therapist variables could prove difficult. While not expected, cyber sickness could be an additional challenge that could affect the patient’s ability to complete the VR sessions (Weech et al., 2019). This could hinder the patient’s ability to complete VR sessions, necessitating withdrawal from the study. Additionally, technical issues and the need for continual software updates represent logistical challenges. To mitigate this, the research team maintains an ongoing collaboration with the VR-CBT software provider, CleVR, to ensure the utilization of up-to-date and optimally functioning VR sets and modules. Another challenge stems from the relatively early stages of VR adoption in clinical forensic practice. Implementation hurdles include therapists’ limited confidence and self-efficacy in using VR, along with practical resource constraints like time and technical support (Kouijzer et al., 2023). Therapists with limited VR experience in this study may hesitate to involve patients and may not fully realize its potential.

### 3.4 Ethical considerations

The study has been approved by the Medical Research Ethics Committee (MREC) of the Amsterdam University Medical Center (UMC), confirming its alignment with ethical standards. All participants will be given a detailed explanation about the study and are required to give written informed consent. Care will be taken to ensure that the VR environment does not trigger excessive stress or other adverse reactions in participants. In the case of adverse effects, the protocol outlines steps for discontinuing the intervention and providing appropriate care. Data confidentiality and participant anonymity will be maintained throughout the study.

### 3.5 Future directions

Given the novel nature of this study, the findings will have implications for future research and clinical practice in forensic outpatients. Should the VR condition prove to be more effective than the standard Re-ART, more robust studies must be conducted to corroborate these findings. Given the heterogeneity of forensic patients and the transdiagnostic focus of this study, future research could investigate the utility of VR in different subgroups to gain a better understanding of how and for whom VR can be valuable. The inclusion of secondary outcome measures and moderators in the study might provide initial insights into this. Furthermore, future studies should examine the use of VR in other forensic populations, such as sexual offenders, intimate partner violence offenders, and property offenders. Moreover, as VR technology continues to evolve, research will need to keep pace, examining the impact of new VR capabilities on treatment outcomes.

## 4 Conclusion

The RCT as described in the study protocol aims to provide valuable data on the effectiveness of incorporating VR into aggression regulation treatment for forensic outpatients. If successful, this study could represent a significant step forward in the use of technology-enhanced interventions in forensic outpatients. Given the high societal costs of aggressive and violent behavior, any improvement in treatment effectiveness has the potential for significant societal impact.

## Ethics statement

The studies involving humans were approved by the Medical Research Ethics Committees (MREC) of the Amsterdam University Medical Center (UMC). The studies were conducted in accordance with the local legislation and institutional requirements. The participants provided their written informed consent to participate in this study.

## Author contributions

BW: Conceptualization, Investigation, Methodology, Visualization, Writing – original draft. JH: Conceptualization, Investigation, Methodology, Supervision, Writing – review & editing. LH: Conceptualization, Investigation, Methodology, Supervision, Writing – review & editing.

## Footnotes

^1^ https://clevr.net/en/index.html

^2^ https://screamingbee.com/Docs/MorphVOXPro/MorphDocIntro
